# Supplementary material for: PD-L1+ Lymphocytes Are Associated with CD4+, Foxp3+CD4+, IL17+CD4+ T Cells and Subtypes of Macrophages in Resected Early-Stage Non-Small Cell Lung Cancer
Source: Int J Mol Sci. 2024 Oct 9;25(19):10827. doi: 10.3390/ijms251910827 (PMC11477418; doi:10.3390/ijms251910827)
Supplement: Supplementary file 1 [file ijms-25-10827-s001.zip › Table S1.docx]

**Table S1.** Association between positive or negative PD-L1 expression evaluated by TPS, IC, CPS and high or low immune cells infiltration in tumour islets and stroma.

|  |  | PD-L1 TPS | |  | PD-L1 IC | |  | PD-L1 CPS | |  |
| --- | --- | --- | --- | --- | --- | --- | --- | --- | --- | --- |
|  |  | negative | positive | *p* | negative | positive | *p* | negative | positive | *p* |
| CD8^+^ T cells n (%) |  |  |  |  |  |  |  |  |  |  |
| Islets | Low | 27 (79.4) | 7 (20.6) | 0.198 | 24 (70.6) | 10 (29.4) | 0.263 | 24 (70.6) | 10 (35.7) | 0.119 |
|  | High | 25 (65.8) | 13 (34.2) |  | 22 (57.9) | 16 (42.1) |  | 20 (52.6) | 18 (64.3) |  |
| Stroma | Low | 24 (70.6) | 10 (29.4) | 0.77 | 22 (64.7) | 12 (35.3) | 0.891 | 22 (64.7) | 12 (35.3) | 0.554 |
|  | High | 28 (73.7) | 10 (26.3) |  | 24 (63.2) | 14 (36.8) |  | 22 (57.9) | 16 (42.1) |  |
| CD4^+^ T cells n (%) |  |  |  |  |  |  |  |  |  |  |
| Islets | Low | 26 (74.3) | 9 (25.7) | 0.704 | 23 (65.7) | 12 (34.3) | 0.754 | 22 (62.9) | 13 (37.1) | 0.768 |
|  | High | 26 (70.3) | 11 (29.7) |  | 23 (62.2) | 14 (37.8) |  | 22 (59.5) | 15 (40.5) |  |
| Stroma | Low | 27 (77.1) | 8 (22.9) | 0.365 | 26 (74.3) | 9 (25.7) | 0.074 | 25 (71.4) | 10 (28.6) | 0.081 |
|  | High | 25 (67.6) | 12 (32.4) |  | 20 (54.1) | 17 (45.9) |  | 19 (51.4) | 18 (48.6) |  |
| Foxp3^+^CD4^+^ T cells n (%) |  |  |  |  |  |  |  |  |  |  |
| Islets | Low | 28 (77.8) | 8 (22.2) | 0.293 | 28 (77.8) | 8 (22.2) | 0.014 | 26 (72.2) | 10 (27.8) | 0.053 |
|  | High | 24 (66.7) | 12 (33.3) |  | 18 (50) | 18 (50) |  | 18 (50) | 18 (50) |  |
| Stroma | Low | 27 (75.0) | 9 (25) | 0.599 | 25 (69.4) | 11 (30.6) | 0.326 | 24 (66.7) | 12 (33.3) | 0.334 |
|  | High | 25 (69.4) | 11 (30.6) |  | 21 (58.3) | 15 (41.7) |  | 20 (55.6) | 16 (44.4) |  |
| IL-17A^+^CD4^+^ T cells n (%) |  |  |  |  |  |  |  |  |  |  |
| Islets | Low | 22 (84.6) | 4 (15.4) | 0.078 | 17 (65.4) | 9 (34.6) | 0.843 | 17 (65.4) | 9 (34.6) | 0.576 |
|  | High | 30 (65.2) | 16 (34.8) |  | 29 (63) | 17 (37) |  | 27 (58.7) | 19 (41.3) |  |
| Stroma | Low | 19 (59.4) | 13 (40.6) | 0.029 | 15 (46.9) | 17 (53.1) | 0.007 | 14 (43.8) | 18 (56.3) | 0.007 |
|  | High | 33 (52.5) | 7 (17.5) |  | 31 (77.5) | 9 (22.5) |  | 30 (75) | 10 (25) |  |
| M1 macrophages n (%) |  |  |  |  |  |  |  |  |  |  |
| Islets | Low | 26 (72.2) | 10 (50) | 1 | 24 (66.7) | 12 (33.3) | 0.624 | 22 (61.1) | 14 (38.9) | 1 |
|  | High | 26 (72.2) | 10 (50) |  | 22 (61.1) | 14 (38.9) |  | 22 (61.1) | 14 (38.9) |  |
| Stroma | Low | 24 (68.6) | 11 (31.4) | 0.501 | 19 (54.3) | 27 (73.0) | 0.099 | 18 (51.4) | 17 (48.6) | 0.101 |
|  | High | 28 (75.7) | 9 (24.3) |  | 16 (45.7) | 10 (38.5) |  | 26 (70.3) | 11 (29.7) |  |
| M2 macrophages n (%) |  |  |  |  |  |  |  |  |  |  |
| Islets | Low | 17 (77.3) | 18 (78.3) | 0.936 | 17 (77.3) | 5 (22.7) | 0.936 | 16 (72.7) | 6 (27.3) | 0.928 |
|  | High | 5 (22.7) | 5 (21.7) |  | 18 (78.3) | 5 (21.7) |  | 17 (73.9) | 6 (26.1) |  |
| Stroma | Low | 17 (81) | 4 (19) | 0.729 | 16 (76.2) | 5 (23.8) | 1 | 15 (71.4) | 6 (28.6) | 0.787 |
|  | High | 18 (75) | 6 (25) |  | 19 (79.2) | 5 (20.8) |  | 18 (75) | 6 (25) |  |

*p<0.05 chi-square (χ2) test.
