# Supplementary material for: PD-L1+ Lymphocytes Are Associated with CD4+, Foxp3+CD4+, IL17+CD4+ T Cells and Subtypes of Macrophages in Resected Early-Stage Non-Small Cell Lung Cancer
Source: Int J Mol Sci. 2024 Oct 9;25(19):10827. doi: 10.3390/ijms251910827 (PMC11477418; doi:10.3390/ijms251910827)
Supplement: Supplementary file 1 [file ijms-25-10827-s001.zip › Table S2.docx]

**Table S2.** Correlation between PD-L1^+^ lymphocytes and tumour infiltrating immune cells.

|  | PD-L1^+^ lymphocytes | | | |
| --- | --- | --- | --- | --- |
|  | islets | | stroma | |
|  | r | *p* | r | *p* |
| CD8^+^ T cells |  |  |  |  |
| Islets | 0.229 | 0.053 | 0.152 | 0.201 |
| Stroma | 0.124 | 0.297 | -0.010 | 0.931 |
| CD4^+^ T cells |  |  |  |  |
| Islets | 0.248 | 0.036 | 0.166 | 0.164 |
| Stroma | 0.343 | 0.003 | 0.362 | 0.002 |
| Foxp3^+^CD4^+^T cells |  |  |  |  |
| Islets | 0.376 | 0.001 | 0.447 | 0.000 |
| Stroma | 0.207 | 0.081 | 0.144 | 0.228 |
| IL-17A^+^CD4^+^ T cells |  |  |  |  |
| Islets | 0.303 | 0.01 | 0.269 | 0.022 |
| Stroma | -0.020 | 0.866 | -0.038 | 0.751 |
| M1 macrophages |  |  |  |  |
| Islets | -0.096 | 0.424 | -0.155 | 0.192 |
| Stroma | 0.096 | 0.421 | 0.057 | 0.632 |
| M2 macrophages |  |  |  |  |
| Islets | 0.248 | 0.101 | 0.206 | 0.174 |
| Stroma | 0.375 | 0.011 | 0.267 | 0.077 |

*p* values are from Spearman rank test.
