# Supplementary material for: PD-L1+ Lymphocytes Are Associated with CD4+, Foxp3+CD4+, IL17+CD4+ T Cells and Subtypes of Macrophages in Resected Early-Stage Non-Small Cell Lung Cancer
Source: Int J Mol Sci. 2024 Oct 9;25(19):10827. doi: 10.3390/ijms251910827 (PMC11477418; doi:10.3390/ijms251910827)
Supplement: Supplementary file 1 [file ijms-25-10827-s001.zip › Table S3.docx]

**Table S3.** Associations between PD-L1 expression level (positive or negative) and tumour immune phenotype.

|  | TPS | | | IC | | | CPS | | |
| --- | --- | --- | --- | --- | --- | --- | --- | --- | --- |
|  | negative | positive | *p* | negative | positive | *p* | negative | positive | *p* |
| CD8^+^ n (%) |  |  |  |  |  |  |  |  |  |
| Desert | 14 (77.8) | 4 (22.2) | 0.425 | 12 (66.7) | 6 (33.3) | 0.470 | 12 (66.7) | 6 (33.3) | 0.294 |
| Excluded | 13 (81.3) | 3 (18.8) |  | 12 (75) | 4 (25) |  | 12 (75) | 4 (25) |  |
| Inflamed | 25 (65.8) | 13 (34.2) |  | 22 (57.9) | 16 (42.1) |  | 20 (52.6) | 18 (47.4) |  |
| CD4^+^ n (%) |  |  |  |  |  |  |  |  |  |
| Desert | 13 (76.5) | 4 (23.5) | 0.894 | 12 (70.6) | 5 (29.4) | 0.803 | 12 (70.6) | 5 (24.4) | 0.66 |
| Excluded | 13 (72.2) | 5 (27.8) |  | 11 (61.1) | 7 (38.9) |  | 10 (55.6) | 8 (44.4) |  |
| Inflamed | 26 (70.3) | 11 (29.7) |  | 23 (62.2) | 14 (37.8) |  | 22 (59.5) | 15 (40.5) |  |
| Foxp3^+^CD4^+^ n (%) |  |  |  |  |  |  |  |  |  |
| Desert | 19 (79.2) | 5 (20.8) | 0.555 | 19 (79.2) | 5 (20.8) | 0.058 | 18 (75) | 6 (25) | 0.138 |
| Excluded | 9 (75) | 3 (25) |  | 9 (75) | 3 (25) |  | 8 (66.7) | 4 (33.3) |  |
| Inflamed | 24 (66.7) | 12 (33.3) |  | 18 (50) | 18 (50) |  | 18 (50) | 18 (50) |  |
| IL17A^+^CD4^+^ n (%) |  |  |  |  |  |  |  |  |  |
| Desert | 9 (69.2) | 4 (30.8) | 0.034 | 6 (46.2) | 7 (53.8) | 0.122 | 6 (46.2) | 7 (53.8) | 0.109 |
| Excluded | 13 (100) | 0 (0) |  | 11 (84.6) | 2 (15.4) |  | 11 (84.6) | 2 (15.4) |  |
| Inflamed | 30 (65.2) | 16 (34.8) |  | 29 (63) | 17 (37) |  | 27 (58.7) | 19 (41.3) |  |
| M1 macrophages n (%) |  |  |  |  |  |  |  |  |  |
| Desert | 17 (73.9) | 6 (26.1) | 0.956 | 14 (60.9) | 9 (39.1) | 0.558 | 13 (56.5) | 10 (43.5) | 0.727 |
| Excluded | 9 (69.2) | 4 (30.8) |  | 10 (76.9) | 3 (23.1) |  | 9 (69.2) | 4 (30.8) |  |
| Inflamed | 26 (72.2) | 10 (27.8) |  | 22 (61.1) | 14 (38.9) |  | 22 (61.1) | 14 (38.9) |  |
| M2 macrophages n (%) |  |  |  |  |  |  |  |  |  |
| Desert | 11 (91.7) | 1 (8.3) | 0.205 | 10 (83.3) | 2 (16.7) | 0.753 | 10 (83.3) | 2 (16.7) | 0.503 |
| Excluded | 6 (60) | 4 (40) |  | 7 (70) | 3 (30) |  | 6 (60) | 4 (40) |  |
| Inflamed | 18 (78.3) | 5 (21.7) |  | 18 (78.3) | 5 (21.7) |  | 17 (73.9) | 6 (26.1) |  |

*p* values are from chi-square (χ2) test.
