# Supplementary material for: PD-L1+ Lymphocytes Are Associated with CD4+, Foxp3+CD4+, IL17+CD4+ T Cells and Subtypes of Macrophages in Resected Early-Stage Non-Small Cell Lung Cancer
Source: Int J Mol Sci. 2024 Oct 9;25(19):10827. doi: 10.3390/ijms251910827 (PMC11477418; doi:10.3390/ijms251910827)
Supplement: Supplementary file 1 [file ijms-25-10827-s001.zip › Table S4.docx]

**Table S4.** Association between PD-L1^+^ expression level (low or high) and immune phenotypes.

|  | TPS | | | IC | | | CPS | | |
| --- | --- | --- | --- | --- | --- | --- | --- | --- | --- |
|  | low | high | *p* | low | high | *p* | low | high | *p* |
| CD8^+^ n (%) |  |  |  |  |  |  |  |  |  |
| Desert | 18 (100) | 0 (0) | 0.408 | 14 (77.8) | 4 (22.2) | 0.147 | 13 (72.2) | 5 (27.8) | 0.509 |
| Excluded | 15 (93.8) | 1 (6.3) |  | 16 (100) | 0 (0) |  | 14 (87.5) | 2 (12.5) |  |
| Inflamed | 34 (89.5) | 4 (10.5) |  | 32 (87.2) | 6 (15.8) |  | 27 (71.1) | 11 (28.9) |  |
| CD4^+^ n (%) |  |  |  |  |  |  |  |  |  |
| Desert | 17 (100) | 0 (0) | 0.59 | 15 (88.2) | 2 (11.8) | 1 | 14 (82.4) | 3 (17.6) | 0.771 |
| Excluded | 17 (94.4) | 1 (5.6) |  | 16 (88.9) | 2 (11.1) |  | 13 (72.2) | 5 (27.8) |  |
| Inflamed | 33 (89.2) | 4 (10.8 |  | 31 (83.8) | 6 (16.2) |  | 27 (73) | 10 (27) |  |
| Foxp3^+^CD4^+^ n (%) |  |  |  |  |  |  |  |  |  |
| Desert | 23 (95.8) | 1 (4.2) | 0.563 | 24 (100) | 0 (0) | 0.002 | 22 (91.7) | 2 (8.3) | 0.024 |
| Excluded | 12 (100) | 0 (0) |  | 12 (100) | 0 (0) |  | 10 (83.3) | 2 (16.7) |  |
| Inflamed | 32 (88.9) | 4 (11.1) |  | 26 (72.2) | 10 (27.8) |  | 22 (61.1) | 14 (38.9) |  |
| IL17A^+^CD4^+^ n (%) |  |  |  |  |  |  |  |  |  |
| Desert | 13 (100) | 0 (0) | 0.513 | 9 (62.2) | 4 (30.8) | 0.072 | 8 (61.5) | 5 (38.5) | 0.041 |
| Excluded | 13 (100) | 0 (0) |  | 13 (100) | 0 (0) |  | 13 (100) | 0 (0) |  |
| Inflamed | 41 (89.1) | 5 (10.9) |  | 40 (87) | 6 (13) |  | 33 (71.7) | 13 (28.3) |  |
| M1 macrophages n (%) |  |  |  |  |  |  |  |  |  |
| Desert | 22 (95.7) | 1 (4.3) | 0.57 | 18 (78.3) | 5 (21.7) | 0.205 | 18 (78.3) | 5 (21.7) | 0.586 |
| Excluded | 13 (100) | 0 (0) |  | 13 (100) | 0 (0) |  | 11 (84.6) | 2 (15.4) |  |
| Inflamed | 32 (88.9) | 4 (11.1) |  | 31 (86.1) | 5 (13.9) |  | 25 (69.4) | 11 (30.6) |  |
| M2 macrophages n (%) |  |  |  |  |  |  |  |  |  |
| Desert | 12 (100) | 0 (0) | 0.466 | 12 (100) | 0 (0) | - | 11 (91.7) | 1 (8.3) | 0.726 |
| Excluded | 9 (90) | 1 (10) |  | 10 (100) | 0 (0) |  | 8 (80) | 2 (20) |  |
| Inflamed | 22 (95.7) | 1 (4.3) |  | 23 (100) | 0 (0) |  | 20 (87) | 3 (13) |  |

*p* values are from chi-square (χ2) test.
