# Supplementary material for: PD-L1+ Lymphocytes Are Associated with CD4+, Foxp3+CD4+, IL17+CD4+ T Cells and Subtypes of Macrophages in Resected Early-Stage Non-Small Cell Lung Cancer
Source: Int J Mol Sci. 2024 Oct 9;25(19):10827. doi: 10.3390/ijms251910827 (PMC11477418; doi:10.3390/ijms251910827)
Supplement: Supplementary file 1 [file ijms-25-10827-s001.zip › Table S5.docx]

**Table S5.** Association between PD-L1^+^ lymphocytes infiltration and immune phenotypes (based on T cell infiltration).

|  | PD-L1^+^ lymphocytes in islets | | PD-L1^+^ lymphocytes in stroma | |
| --- | --- | --- | --- | --- |
|  | median (range) | *p* | median (range) | *p* |
| CD8^+^ |  |  |  |  |
| Desert | 3.5 (0-37) | 0.294 | 55.5 (0-433) | 0.484 |
| Excluded | 2 (0-116) |  | 23 (0-836) |  |
| Inflamed | 9 (0-121) |  | 70.5 (0-539) |  |
| CD4^+^ |  |  |  |  |
| Desert | 0 (0-121) | 0.191 | 10 (0-433) | 0.24 |
| Excluded | 3.5 (0-54) |  | 32.5 (0-390) |  |
| Inflamed | 13 (0-116) |  | 76 (0-836) |  |
| Foxp3^+^CD4^+^ |  |  |  |  |
| Desert | 0 (0-54) | 0.005 | 23.5 (0-262) | 0.006 |
| Excluded | 7 (0-121) |  | 11.5 (0-390) |  |
| Inflamed | 14.5 (0-116) |  | 86 (0-836) |  |
| IL17A^+^CD4^+^ |  |  |  |  |
| Desert | 3 (0-37) | 0.018 | 136 (0-521) | 0.011 |
| Excluded | 0 (0-31) |  | 9 (0-90) |  |
| Inflamed | 9 (0-121) |  | 65.5 (0-836) |  |

*p* values are from Kruskal–Wallis test.
